# Maximized response by structural optimization of soft elastic composite systems

**DOI:** 10.1093/pnasnexus/pgae353

**Published:** 2024-08-21

**Authors:** Lukas Fischer, Andreas M Menzel

**Affiliations:** Institut für Physik, Otto-von-Guericke-Universität Magdeburg, Universitätsplatz 2, Magdeburg 39106, Germany; Institut für Physik, Otto-von-Guericke-Universität Magdeburg, Universitätsplatz 2, Magdeburg 39106, Germany

**Keywords:** soft magnetic functional materials, composite materials, actuation, material optimization

## Abstract

Soft actuators triggered in a wire—and contactless way advance soft robotics, for instance, concerning microsurgical perspectives. For optimal performance in this and other contexts, maximized stimuli-responsiveness is frequently desirable. We demonstrate on the example of soft magnetoelastic systems how analytical theoretical measures in combination with computer simulations provide tools to develop optimized components. To enhance the overall macroscopic response, we adjust microstructural properties. Our strategy guides us towards ideally structured soft materials that can be fabricated using modern technologies.

**Significance Statement**

New technological developments, for example 3D printing, increasingly allow to fabricate soft elastic composite materials containing discrete sites of functionalization at prescribed positions. The full potential of such materials will only be exploited, if we manage to optimize the discrete internal positioning. Combining analytical theory and computational tools, we introduce an efficient procedure to perform such optimization on a representative example system. We demonstrate how different optimized discrete arrangements emerge depending on the requested overall mechanical response. This will substantially advance the capabilities of these soft functionalized materials. Together, our approach and results will be of interest to a community who searches to advance the properties of matter in general.

## Introduction

As the backbone of soft robotics, soft actuators have attracted significant interest (1–3). They feature many advantages compared to conventional solutions, such as light weight (2, 4) and—due to their mechanical softness—improved human-robot collaboration and handling of fragile items (1–3). Also, they are relatively inexpensive (4–6) and compact (3, 5). These materials show an active deformation or stress generation in response to external stimuli. Many triggers can be used, for example heat, light, chemicals, electric, or magnetic fields (3, 6–13). Typically, magnetic fields do not interact severely with biological tissue, which is important for biological and medical applications (5–8, 14). Consequently, we focus on soft magnetoelastic systems, that is, materials consisting of a soft, often polymeric, matrix with embedded magnetic or magnetizable particles (6, 10, 15–19). When external magnetic fields are applied, induced magnetic interactions between the inclusions cause an overall, macroscopic deformation (magnetostriction) and change in macroscopic behavior. These materials are discussed as candidates for artificial muscles (11, 20) of reversible deformability (21) and adaptive dampers or vibration absorbers (22–24). The latter is due to magnetically tunable rheological properties—the so-called magnetorheological (MR) effect (15, 21, 25–29).

So far, most investigations have concentrated on corresponding materials featuring rather randomized internal particle arrangements (15, 18, 30–32). As one variation, anisotropic and chain-like structures are generated in strong uniform external magnetic fields (18, 28, 30, 33, 34). However, in general, such types of spatial arrangement will not lead to an optimized deformational response.

The advent and development of new routes of sample preparation will in the future allow for a significantly improved targeted placement of magnetizable inclusions in elastic carrier media. Accordingly, spatial arrangements tailored to the requested purpose are realized. Internal structures are optimized for maximized deformational response. Promising example techniques include 3D printing (12, 13, 29, 35–37), structuring by magnetic fields (38), sequential photopolymerization (39), acoustic holography (40), layerwise polymerization combined with particle placement by molds or by hand (41, 42), and wax-cast molding (43). We here introduce efficient means to determine the structure of optimized spatial arrangements of inclusions that afterward can be realized by the listed experimental methods.

Concerning the types of inclusions considered, we note that our approach does not distinguish between individual particles or agglomerates of these, for instance, in a drop of magnetic fluid. Yet, we require the condition of well-separated inclusions. Besides, due to the generic nature of our approach, the overall size of the system is not preset. Only the size of the inclusions relative to the overall size needs to be small. Therefore, as long as this condition is satisfied, even macroscopic inclusions can be described by our approach.

## Results

### Model for calculating the magnetostrictive effects

Our approach is based on analytical theory, which serves as an input for our computational optimization of soft magnetoelastic materials. Performing analytical calculations on finite-sized elastic systems is a challenge. We had managed to calculate explicitly the deformation of a homogeneous, isotropic, linearly elastic sphere in response to an internal force distribution (44). The shear modulus *μ* characterizes the elastic stiffness of the sphere and the Poisson ratio ν∈(−1,1/2] its compressibility. Materials of ν=1/2 are incompressible, for ν<1/2 they are compressible, and for ν<0 auxetic. For small deformations, nonlinear functionals of the elastic energy can usually be expanded into a power series. There are generally only two different possible types of quadratic terms that may emerge (45). They represent linearly elastic behavior. Consequently, analyzing the behavior in terms of linear elasticity forms a reasonable and generic starting point.

**Fig. 1.**
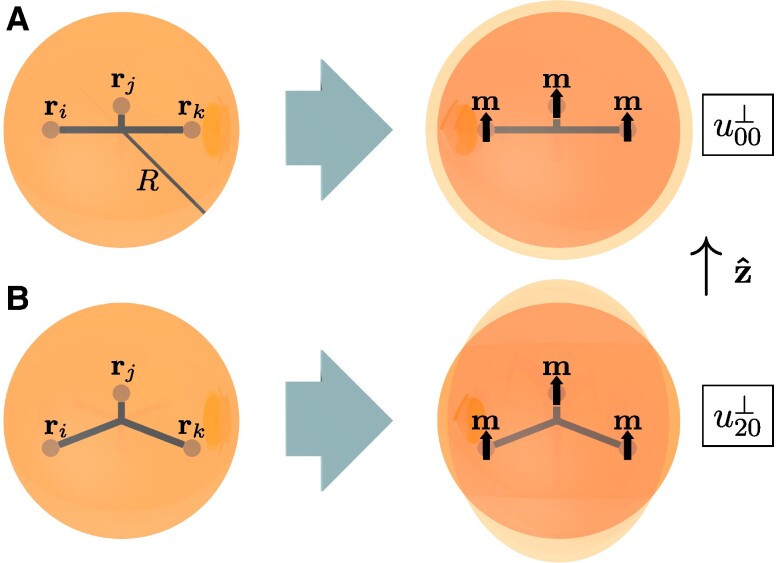
Illustration of the modes of deformation $u_{00}^{⊥}$ in A) and $u_{20}^{⊥}$ in B). In the undeformed spherical ground states on the left, we here indicate three magnetizable inclusions as smaller spheres at positions $r_{i}$, $r_{j}$, and $r_{k}$. Upon magnetization (right), their interacting magnetic dipole moments $m∥\hat{z}$ induce deformations of the whole sphere, implying changes in volume ($u_{00}^{⊥}$) and elongation along $\hat{z}$ relative to lateral contraction ($u_{20}^{⊥}$). Deformed states are illustrated in lighter color (orange) with amplitudes not to scale.

We first consider spherical model systems containing *N* magnetic force centers. Thus, we explicitly include the boundaries of the system into our consideration. Induced changes in volume and shape, that is, magnetoelastic actuations, are quantified using the radial outward displacement field of the surface points. We expand this field into spherical harmonics (44). A first expansion coefficient $u_{00}^{⊥}$ quantifies the overall induced change in volume, see Fig. 1A. A second expansion coefficient $u_{20‘}^{⊥}$ quantifies elongation along a certain axis $\hat{z}$ relative to lateral contraction, see Fig. 1B. We here present explicit analytical expressions forming the basis of our optimization. Point-like force centers of forces $F_{i}$ acting at positions $r_{i}$, i=1,…,N, with the origin of our coordinate frame positioned at the center of the sphere of radius *R*, imply (46)


(1) $$u_{00}^{⊥}=\sum_{i=1}^{N}F_{i}\cdot r_{i}\frac{1-2\nu }{1+\nu }\frac{1}{2\mu R^{2}}\sqrt{\frac{1}{4\pi }},$$

(2) $$\begin{array}{rl} u_{20}^{⊥} & =\sum_{i=1}^{N}\frac{\sqrt{5/4\pi }}{4\mu R^{2}(7+5\nu )}[(-F_{i}\cdot {\underline{\delta }}^{⊥}\cdot r_{i}+2F_{i}\cdot {\underline{\delta }}^{∥}\cdot r_{i})\left(7+2\nu \right)\\ & \;+3\frac{r_{i}^{3}}{R^{2}}\left(2\nu F_{i}\cdot {\underline{\delta }}^{⊥}\cdot {\hat{r}}_{i}-(7-6\nu )F_{i}\cdot {\underline{\delta }}^{∥}\cdot {\hat{r}}_{i}+F_{i}\cdot {\hat{r}}_{i}{\hat{r}}_{i}\cdot {\underline{\delta }}^{∥}\cdot {\hat{r}}_{i}(7-10\nu )\right)]. \end{array}$$

Here, ${\hat{r}}_{i}=r_{i}/r_{i}$, $r_{i}=|r_{i}|$, ${\underline{\delta }}^{∥}=\hat{z}\hat{z}$, ${\underline{\delta }}^{⊥}=1-{\underline{\delta }}^{∥}$, $\hat{z}\hat{z}$ is a dyadic product, and 1 the unit matrix.

Each point-like force center represents a magnetizable inclusion. We require a certain distance between any two force centers $|r_{i}-r_{j}|>0.12R$, and a minimal distance from the boundary $r_{i}<0.94R$, i≠j=1,…,N. These settings (and the overall number of inclusions *N*) affect the resulting structures. The chosen values favor a representation using magnetic dipolar interactions and linear elasticity. Considering saturated magnetization by strong external homogeneous magnetic fields along $\hat{z}$, we obtain (47)


(3) Fi=−∑j=1j≠iN3μ0m2[5r^ij(m^⋅r^ij)2−r^ij−2m^(m^⋅r^ij)]4πrij4.

Here, $m=m\hat{m}$ is the identical magnetic dipole moment for all inclusions, where $\hat{m}=\hat{z}$, $r_{ij}=r_{i}-r_{\;j}$, $r_{ij}=|r_{ij}|$, ${\hat{r}}_{ij}=r_{ij}/r_{ij}$, and $\mu_{0}$ denotes the magnetic vacuum permeability. After rescaling, we measure lengths in units of *R* and the relative strength of the magnetic dipolar interaction by a nondimensional number $3\mu_{0}m^{2}/4\pi \mu R^{6}$. The latter is set to a realistic value of $5.4\times 10^{-8}$ (44) for μ≈1.67kPa (48–51), inclusions of radius r=0.02R, and a saturation magnetization of $518\;\mathrm{kA}\;m^{-1}$ as for ${\text{Fe}}_{3}{\text{O}}_{4}$ (52).

As a central benefit, we can now determine the maxima of the analytical expressions in Eqs. 1 and 2 as a function of the positions. That is, we find the largest degrees of deformation associated with the amplitudes $\pm u_{00}^{⊥}$ or $\pm u_{20}^{⊥}$. Optimization is performed as a function of the internal structural arrangements. We recall that the number of inclusions is fixed during this procedure.

Because of the many degrees of freedom $r_{i}$, i=1,…,N, we resort to numerical procedures to achieve optimization. We employ simulated annealing (SA) (53–56) with modifications (57) and adjusted parameter settings, see the Supplementary material. Typically, this method is used to minimize energies. Here, instead, we insert the right-hand sides of Eqs. 1 and 2 to maximize the deformations induced by the magnetized inclusions. The extrema are found using the locations of the *N* inclusions as degrees of freedom, maintaining the above-mentioned constraints. Accordingly, we transfer this well-established method to the field of material optimization.

### Resulting materials of maximized actuation

#### Linear actuation

First, we maximize the relative contraction along the magnetic field direction, referring to linear actuators or artificial muscles. We thus minimize $u_{20}^{⊥}$ for N=1,000 magnetizable inclusions, assuming incompressibility (48, 49, 51). The resulting optimized structure achieves an approximately 68% higher degree of deformation $u_{20}^{⊥}$ when compared to realizations of regular lattice structures (44).

**Fig. 2.**
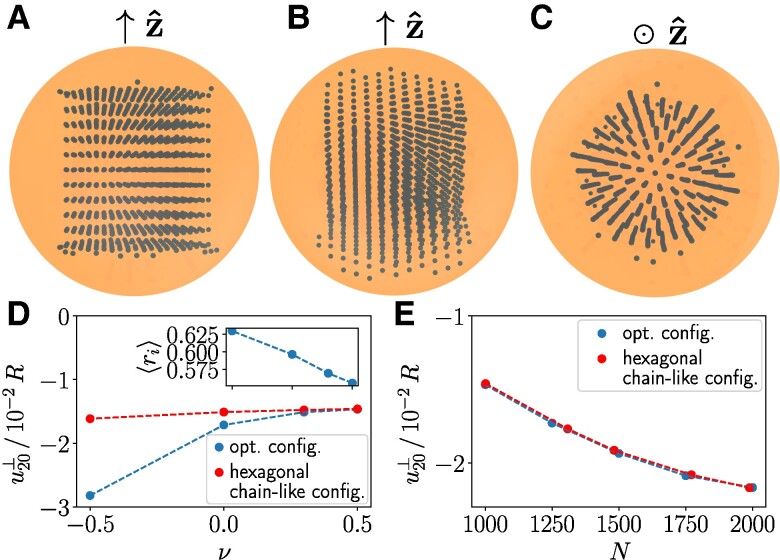
Maximized relative contraction along the magnetization direction $\hat{z}$, minimizing $u_{20}^{⊥}$. A–C) Resulting optimized configuration of magnetizable inclusions (“opt. config.”) inside an incompressible elastic sphere of radius *R* (orange) for N=1,000. A) Side view. B) Tilted top view. C) Top view. D) Degree of deformation as a function of the Poisson ratio *ν*. Inset: average inclusion distance from the center $\left\langle r_{i}\right\rangle$ vs. Poisson ratio (same scale of abscissa as in main plot). E) Variation of the degree of deformation with the inclusion number *N*. In D and E), we further compare deformational results for our optimized structures to those for regular hexagonal chain-like configurations, with dashed lines added as guides to the eye.

Figure 2A–C indicates that the optimal configuration closely resembles a hexagonally arranged chain-like structure. The chains are oriented along the magnetization direction and concentrated towards the center axis. Comparative tests arranging regular structures by hand confirm this picture, see the Supplementary material. Decreasing Poisson ratios increase the magnitudes of deformation, see Fig. 2D. Simultaneously, the inclusions of the optimized structures are shifted closer to the boundary of the sphere, see the inset of Fig. 2D, and deviations from results for regular structures become significant. Increasing the inclusion density mostly enhances the deformational response, see Fig. 2E for ν=1/2. In that case, the regular hexagonal structures perform close to optimally.

Natural muscles often work in counteracting pairs (11). We therefore continue by discussing relative elongations along the magnetization direction, maximizing $u_{20}^{⊥}$. For ν=1/2 and N=1,000, our optimization leads to an increase of approximately 129% when compared to regular structures (44).

**Fig. 3.**
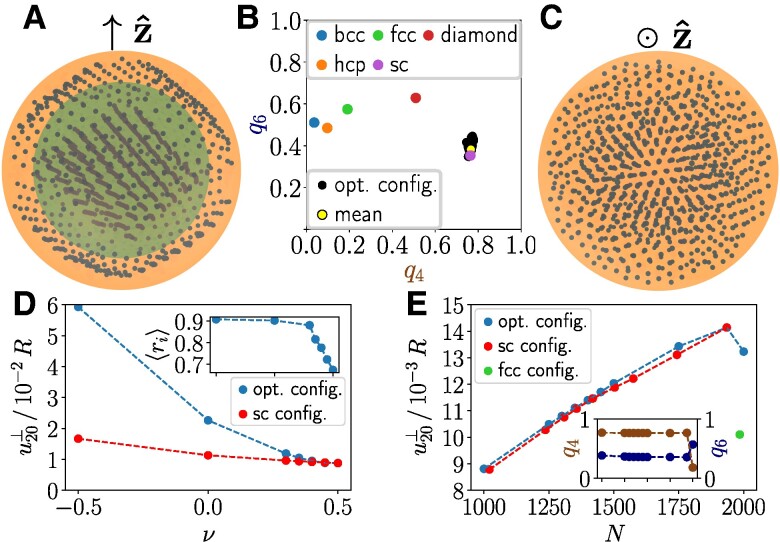
Maximized relative elongation along the magnetization direction $\hat{z}$, maximizing $u_{20}^{⊥}$. A, C) are analogous to Fig. 2. In A), we mark the separation between an inner and an outer part in green (slightly darker color). B) Steinhardt bond orientational order parameters $q_{4}$ and $q_{6}$ for the inner part compared to various regular lattices. D, E) are analogous to Fig. 2, now showing results for appropriately oriented regular simple cubic (sc) configurations. E) The result for a regular face-centered cubic (fcc) structure at N=1,985 is included. Inset: variation of $q_{4}$ (brown, darker color) and $q_{6}$ (blue, lighter color) with *N* (same scale of abscissa as in main plot) indicate a significant change in the optimized texture at N≈2,000.

As Fig. 3A illustrates, the optimized configuration now splits into an inner and an outer part. Both parts contain about the same number of inclusions and also contribute similarly to $u_{20}^{⊥}$. The inner part can be approximated by a regular simple cubic structure, as indicated by Steinhardt bond orientational order parameters (58), see Fig. 3B. Generating an appropriately oriented regular simple cubic configuration by hand over the whole sphere (Supplementary material), the overall degree of deformation $u_{20}^{⊥}$ deviates by only about 0.3%.

When decreasing the Poisson ratio *ν*, the deformational response increases, see Fig. 3D. Simultaneously, the gap in response between optimized and regular simple cubic structure increases. The optimized locations of the inclusions are pushed closer to the spherical surface, see the inset of Fig. 3D.

Again for ν=1/2, Fig. 3E demonstrates that the deformational response mostly increases with increasing number of inclusions. Our regular simple cubic structure remains close to optimal. A disjoint inner and outer part can only be identified for N≲1,400. For N=2,000, the simple cubic configuration is not dense enough and the deformational response decreases. Still, a regular, denser, face-centered cubic (fcc) lattice structure performs significantly worse.

#### Changes in volume

Next, we focus on magnetically induced overall changes in volume, quantified by $u_{00}^{⊥}$. Corresponding systems may prove useful in the design of soft magnetoelastic valves (59) or microfluidic pumps (5). Changes in volume are only possible in compressible elastic materials. For ν<1/2, Eq. 1 demonstrates that materials of different Poisson ratio *ν* all show qualitatively the same dependence of $u_{00}^{⊥}$ on the inclusion configuration. Thus, all of them thus lead to identical optimized structures.

**Fig. 4.**
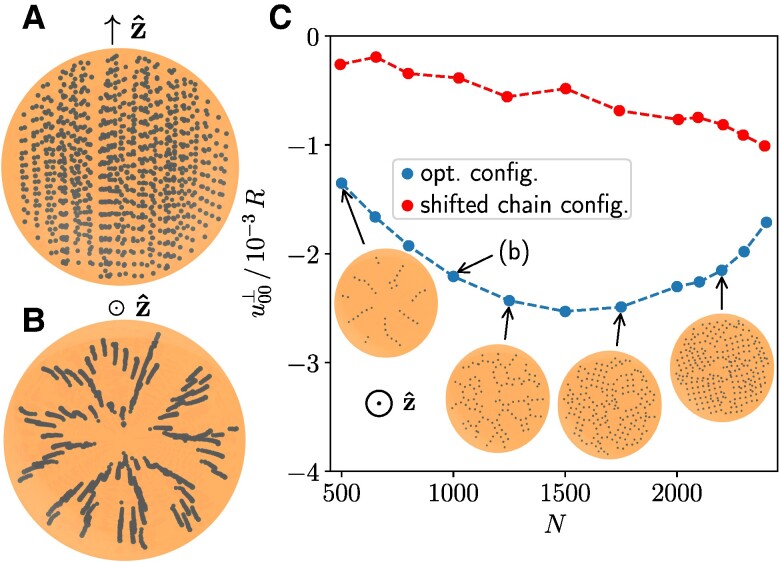
Maximized decrease in total volume, minimizing $u_{00}^{⊥}$. According to Eq. 1, the resulting optimized structures are independent of the Poisson ratio *ν* (here ν=0.3). A) Tilted top view. B) Top view. C) It is analogous to Fig. 2E, supplemented by top views of the mean inclusion positions along each chain-like element for different inclusion numbers *N*.

We first search for a most pronounced induced shrinkage in volume, minimizing $u_{00}^{⊥}$. For N=1,000, this optimized shrinkage increases by 24% in magnitude when compared to regular structures (44). The optimized structure mainly consists of (irregular) chain-like arrangements oriented along the magnetization direction, see Fig. 4A, B. Any two nearest-neighboring chains are shifted relative to each other along their axes by about half a vertical inclusion separation distance (Fig. S2).

With increasing *N*, see Fig. 4C, the arrangements remain predominantly chain-like. However, the chains tend to form square-like lattices. We compare to results for related regular structures, with neighboring chains vertically shifted (Supplementary material). Deviations are significant, see Fig. 4C. Increasing *N* from about 500 to 1,500 raises the magnitude of deformational response, in contrast to the subsequent decrease, thus showing nonmonotonic behavior.

**Fig. 5.**
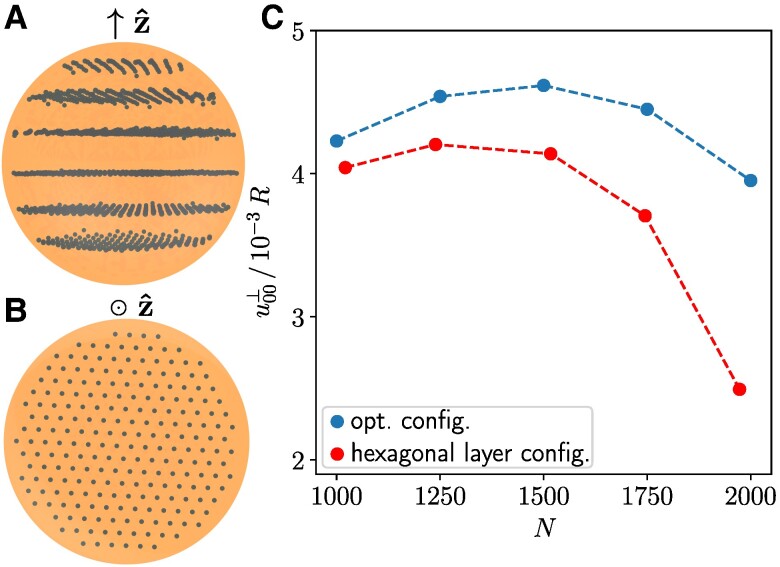
Maximized total increase in volume, maximizing $u_{00}^{⊥}$ (here for ν=0.3). A) Side view. B) Top view, here only displaying the third layer from the bottom in (A). C) is analogous to Fig. 2E.

Lastly, we maximize the induced increase in volume by maximizing $u_{00}^{⊥}$. For N=1,000, our optimized induced expansion is approximately 418% larger when compared to results for regular structures (44). Figure 5A, B indicates the optimized structures to resemble hexagonal arrangements in layers stacked on top of each other. The layer-to-layer distances are much larger than the inter-inclusion distances within each layer, see Fig. 5A, B. Regular hexagonal layer structures generated by hand (Supplementary material) performed notably worse for elevated numbers of inclusions *N*, see Fig. 5C.

### Optimized magnetorheological effects

#### (Positive) magnetorheological effect

Additionally, we optimize the inclusion arrangement to maximize the magnetorheological (MR) effect of the soft magnetoelastic materials, here for prescribed affine deformations. We address cubical systems of side length *a*, maintaining the same conditions as before. The radius of the inclusions is set to r=0.03a, which shifts the nondimensional number $3\mu_{0}m^{2}/4\pi \mu a^{6}$ to approximately $6.2\times 10^{-7}$. To this end, we define $\Delta \mu_{\mathrm{rel}}=(\mu_{\mathrm{magn}}-\mu )/\mu$ as the relative change in shear modulus, where $\mu_{\mathrm{magn}}$ is the overall elastic shear modulus in the magnetized state as measured for our numerical systems.

Our cube is magnetized perpendicular to one of its facets, and we consider incompressible systems. The associated overall energy is given by


(4) E=Edef−∑i,j=1j>iNμ0m2[3(m^⋅r^ij)2−1]4πrij3,

where $E_{\mathrm{def}}$ is the elastic deformation energy followed by the magnetic dipolar interaction energy (47) in the deformed state.

First, we maximize the MR effect when the system is uniaxially elongated along the magnetization direction. That is, $E_{\mathrm{def}}=\frac{3}{2}\mu \delta^{2}a^{3}$ in Eq. 4, where *δ* is the stretching ratio. To calculate $\Delta \mu_{\mathrm{rel}}$, we evaluate the total energy, see Eq. 4, for several values of the stretching ratio *δ* close to the energetic minimum. Afterward, we fit the evaluated values of the total energy by a quadratic function. We determine the minimum of the resulting parabola. It identifies the new equilibrium state under magnetization. Then, the change in the quadratic coefficient of the energy around its minimum from the nonmagnetized to the magnetized state, obtained from the parabolic fit, provides $\Delta \mu_{\mathrm{rel}}$. To fit the parabola, we always calculate the energy at five points around its minimum. We have confirmed that only minor changes (<0.01%) emerge when using 100 points.

**Fig. 6.**
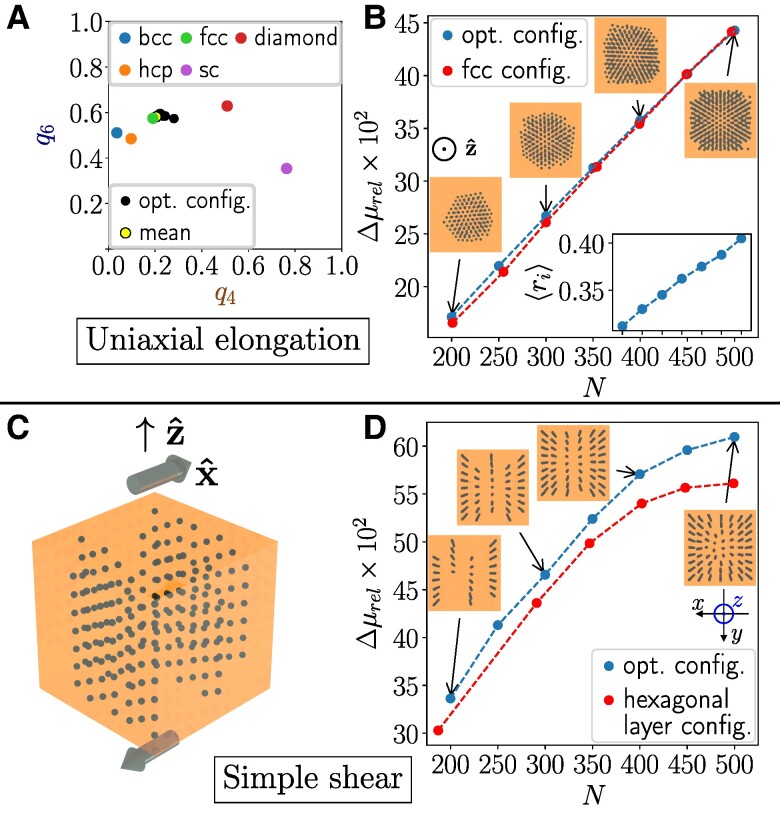
Maximized relative increase in elastic modulus $\Delta \mu_{\mathrm{rel}}$ for cubical systems by magnetization along $\hat{z}$ for A, B) uniaxial elongation, here ν=1/2, and C, D) simple shear. A) Steinhardt bond order parameters for that configuration of N=200 that maximizes $\Delta \mu_{\mathrm{rel}}$. C) Tilted top view. Gray arrows indicate imposed simple shear deformations. B, D) Variation of the relative magnetorheological effect with inclusion number *N*, including top views of the optimized configurations. Inset in B): average distance $\left\langle r_{i}\right\rangle$ from the center of the cube (same scale of abscissa as in main plot).

**Fig. 7.**
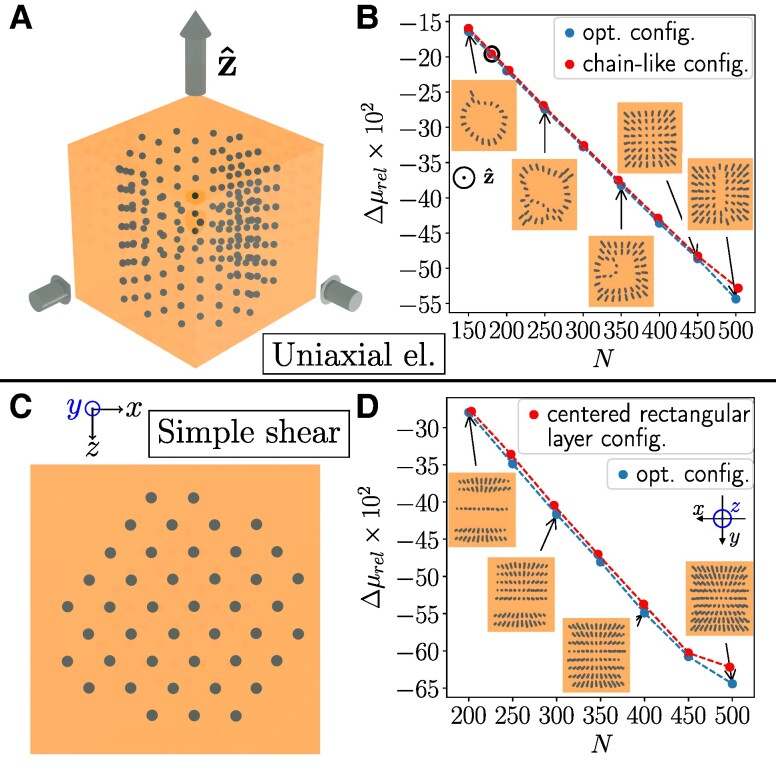
Maximized magnitude of the negative MR effect, that is, strongest relative decrease in elastic modulus $\Delta \mu_{\mathrm{rel}}$ upon magnetization. The illustration is analogous to Fig. 6 for the positive MR effect. Magnetization is imposed along $\hat{z}$. A, B) Uniaxial elongation, here for ν=1/2. C, D) Simple shear. In B), we mark by a black circle the data point for N=180, for which a circular texture is constructed by hand for comparison, see Section IIB of the Supplementary Material. C) Side view of the largest layer of inclusions forming when maximizing the magnitude of the negative MR effect under simple shear for N=200. It corresponds to the bottommost layer in the leftmost inset (top views) in (D).

Our optimization for N=200 reveals a MR effect of $\Delta \mu_{\mathrm{rel}}\approx 17.1\%$. In that case, the optimized structure is reminiscent of an appropriately oriented fcc lattice (Supplementary material), see the Steinhardt bond orientational order parameters in Fig. 6A (an illustration of the imposed deformation is provided in Fig. 7A). Raising *N* fills the cube starting from the center outwards, see the inset of Fig. 6B, with an increasing MR effect. Here, regular structures (Supplementary material) perform comparatively well (within >96%).

For simple shear deformations, $E_{\mathrm{def}}=\frac{1}{2}\mu \gamma^{2}a^{3}$ in Eq. 4, with *γ* as the shear ratio. Shear is imposed within the *xz*-plane, see Fig. 6C. Upon optimization, $\Delta \mu_{\mathrm{rel}}\approx 33.6\%$ for N=200. The optimized structures feature layers in the *yz*-plane of approximately hexagonal structure and of maximized mutual spacing. Increasing *N*, the layers approach each other, see Fig. 6D, with increasing deviations in their performance from regular structures (Supplementary material) (remaining within 90%).

#### Negative magnetorheological effect

The previously considered structures show maximal hardening upon magnetization. Next, we also include results for the opposite scenario, which is significantly less frequently considered (60, 61). It is referred to as negative MR effect, that is, the materials soften upon magnetization. Thus, we now minimize $\Delta \mu_{\mathrm{rel}}$.

Figure 7A depicts the resulting optimized structure for N=200 inclusions and uniaxial elongation, featuring a negative MR effect of $\Delta \mu_{\mathrm{rel}}\approx -22\%$. For N≲200, we observe that the inclusions form chains of a particular arrangement, approximately a circle with an appendage, see the leftmost inset in Fig. 7B. Neighboring chains feature a vertical shift, similar to the configurations that minimize $u_{00}^{⊥}$ from Fig. 4 (see also Fig. S2). With increasing number of inclusions *N*, the optimized arrangements of the chains change. More and more chains form, shifting the overall locations of chains from circular to cubic arrangements, see again the insets of Fig. 7B. Additionally, we infer from Fig. 7B that the MR effect $\Delta \mu_{\mathrm{rel}}$ is almost perfectly proportional to the number of inclusions *N*. For comparison in performance, we construct regular structures by hand using quadratic and circular arrangements of the chains (Supplementary material) with good agreement (>97%) concerning the resulting negative MR effect.

For simple shear deformations, we find a maximized negative MR effect of $\Delta \mu_{\mathrm{rel}}\approx -27.9\%$ for N=200 inclusions as displayed in Fig. 7D. See also Fig. 6C for an illustration of the imposed shear deformations. Here, we observe that our optimized textures can be divided into layers of inclusions oriented parallel to the shear plane. For N=200, the largest such layer is depicted in Fig. 7C. These layers approximately feature centered rectangular structures with edge lengths along the magnetization direction and the direction of shear displacements. The larger edge length is found along the direction of shear displacements. When we increase the number of inclusions *N*, see Fig. 7D, we observe that the number of layers in the optimized arrangements as well as the magnitude of the negative MR effect increase. The results for regular lattice structures created by hand (Supplementary material) show good agreement (>96%).

## Conclusion

In summary, we present a route towards the design of optimized soft elastic composite materials that are structured in a way to maximize the requested overall response. To this end, we have developed and successfully applied corresponding tools. As a result, very efficient schemes of optimization are established and appropriately structured systems are presented.

As examples, we have demonstrated the optimization for soft magnetoelastic systems that respond to external magnetic fields by deformations and changes in mechanical properties. Specifically, we have maximized by adjusting the internal structure the relative elongation and contraction along the magnetization direction, the overall change in volume, and the magnetorheological effect in response to an external magnetic field. Aspects of how the deformational response and the optimized structures depend on the elastic properties of the employed material and on the total number of inclusions are addressed. We have shown that regular arrangements of inclusions do not necessarily maximize the response for finite-sized systems. Future refinements may include adjusted material models, for instance, concerning selected nonlinear elastic models, although linear elastic descriptions represent a substantial initial step of identifying most suitable arrangements (62). Including additional degrees of freedom for optimization, for instance, orientations of rod-shaped inclusions (63), may lead to further improvement.

Our results support the construction of soft components of tailored optimized response in general. Through recent technological developments, requested placement of inclusions in elastic matrices becomes possible (12, 13, 13, 29, 35–43). Applications include, but are not limited to artificial muscles, soft elastic valves and pumps, adaptive dampers, or vibration absorbers. The scheme of optimization that we present can be transferred to various other types of soft responsive composite materials. An immediate example are electrorheological gels and elastomers (50, 64, 65) or thermally actuated systems (66). More generally, whenever it is possible to link the discrete microscopic structure to the overall material behavior, a similar scheme of optimization can be employed to find the structure that maximizes the response. Overall, we support the growing field of tailored soft materials.

## Supplementary Material

pgae353_Supplementary_Data

## Data Availability

The generated data and numerical codes used to derive the results presented herein are published on the repository Zenodo and can be found at https://doi.org/10.5281/zenodo.13549320.
