# Supplementary material for: Maximized response by structural optimization of soft elastic composite systems
Source: PNAS Nexus. 2024 Aug 21;3(9):pgae353. doi: 10.1093/pnasnexus/pgae353 (PMC11382289; doi:10.1093/pnasnexus/pgae353)
Supplement: pgae353_Supplementary_Data [file pgae353_supplementary_data.pdf]

Supplementary material to
"Maximized response by structural optimization of soft elastic composite systems"

Lukas Fischer[1] and Andreas M. Menzel[1]

[1] *Institut für Physik, Otto-von-Guericke-Universität Magdeburg, Universitätsplatz 2, 39106 Magdeburg, Germany*

(Dated: August 30, 2024)

In this supplementary material, we present technical details on the adapted algorithm of simulated annealing (SA) that we employ to adjust the internal structure to optimize a requested overall deformational or mechanical response. Particularly, we list our parameter settings for the algorithm. Furthermore, we provide more background information concerning the regular lattice structures to which we compare the performance of our optimized textures. We list corresponding lattice constants and information on how the number of inclusions N is varied.

I. TECHNICAL DETAILS ON OUR COMPUTATIONAL SCHEME

To optimize the deformational or mechanical response of our magnetoelastic actuator systems, we utilize simulated annealing (SA) with adaptive cooling rates. For this purpose, the algorithm in Ref. 1 is adapted to our situation, see also the main text.

At the beginning of each process of optimization, we initialize the positions of the inclusions at random, unless indicated otherwise. One inclusion is randomly selected at a time. To generate a new trial configuration, its position is modified by a displacement randomly chosen in the range of $[-\delta/2, \delta/2)$ for each direction. This marks one Monte Carlo (MC) step [2], where we measure lengths in units of the radius R of the elastic sphere when optimizing the magnetostrictive effect or in units of side length a of the elastic cube when addressing the magnetorheological (MR) effect. The positional constraints mentioned in the main text are obeyed, that is, minimal distances between the inclusions and from the boundary of the elastic sphere/cube are ensured. Every n_{jump} MC steps, we instead generate a completely new trial position for the selected inclusion, still maintaining the positional constraints. Individual positional changes favorable to the optimization are always accepted. Those implying an opposite trend are only accepted with a certain probability, namely $e^{-\Delta u/T}$, if $\Delta u > 0$. Here, T is a temperature-like variable and Δu marks the difference between the new trial configuration and the previous configuration, where $u = \pm u^{\perp}_{20}/R$ or $u = \pm u^{\perp}_{00}/R$ in our case, depending on the deformational mode to be minimized or maximized in the magnetostrictive case. For the optimization of the MR effect, we instead insert $u = \pm\Delta\mu_{rel}$. Since u is dimensionless by definition, T is also introduced in a dimensionless way. Afterwards, we move on to the next MC step, testing for a new trial configuration.

As our optimization is based on SA, we decrease the temperature T during the course of optimization [3]. Our "cooling schedule" starts from an initial temperature $T = T_i$. We equilibrate the system for a period of N_{eq} MC steps at the current temperature T. Afterwards, we use N_{prod} steps to sample a specific heat-like variable

$$C_V = \frac{\langle u^2\rangle - \langle u\rangle^2}{T^2}, \tag{S1}$$

where $\langle u^2\rangle$ and $\langle u\rangle$ mark the mean squared and mean values of u, see above. Still, the temperature remains fixed during these N_{prod} MC steps. During the next N_{cool} MC steps of our optimization, we reduce the temperature each time before generating a new trial configuration. We decrease the temperature geometrically, that is we set $T = (1-k)T_{old}$, where T_{old} denotes temperature in the previous MC step and k determines the cooling rate.

In our algorithm, the cooling rate for this period is set depending on the specific heat that was evaluated in the previous N_{prod} steps:

$$k = \begin{cases} k_s, & \text{if } C_V \geq C_V^*, \\ k_f, & \text{if } C_V < C_V^*. \end{cases} \tag{S2}$$

Here, we introduced the notations k_s and k_f for slow and fast cooling rates ($k_s \leq k_f$), respectively, and C_V^* as a characteristic threshold value of the specific heat. Consequently, our idea is to cool the system more slowly when more changes are occurring during the process of measuring the specific heat, thus allowing the system more opportunities to keep changing. Otherwise, the system is cooled faster to speed up the optimization. Together, these $N_{eq} + N_{prod} + N_{cool}$ MC steps denote one period of optimization. The same steps are repeated during the next period. Finally, our routine is stopped when no positional changes have been accepted for a number of *patience* periods (during sampling), or when the temperature has decreased to a predetermined final value of T_f.

To summarize, our scheme makes use of the parameters δ, n_{jump}, T_i, N_{eq}, N_{prod}, N_{cool}, k_s, k_f, C_V^*, *patience*, and T_f. All parameter settings that were utilized for the optimization, leading to the structures depicted in Figs. 2A–C, 3A–C of the main text for $N = 1000$ and $\nu = 0.5$, Figs. 4A,B, 5A,B of the main text for $N = 1000$ and $\nu = 0.3$, Figs. 6A,C and 7A,C of the main text for $N = 200$, are listed in Tab. I.

When turning to other values of the Poisson ratio ν or the number of inclusions N, see subfigures B, C, D,

or E of Figs. 2–7 of the main text, we usually keep most of the parameter settings as in Tab. I. It is, however, necessary to rescale the temperature-like variable in the exponents $e^{-\Delta u/T}$, because the typical magnitudes of deformation entering Δu change with ν and N. For simplicity, we rescale initial and final temperatures equally. Therefore, we define $T_i(\nu, N) = T_i\,\alpha(\nu, N)$ and $T_f(\nu, N) = T_f\,\alpha(\nu, N)$, where $\alpha(\nu, N)$ takes care of the required rescaling. We set $\alpha(0.5, 1000) = 1$ when addressing $\pm u_{20}^{\perp}$, $\alpha(0.3, 1000) = 1$ when addressing $\pm u_{00}^{\perp}$, and $\alpha(0.5, 200) = 1$ when addressing the MR effect. The employed values of α and all other adjusted parameter settings, if different from the values shown in Tab. I, when varying ν and N are listed in Tabs. II–VIII.

	δ/R	n_{jump}	T_i	N_{eq}	N_{prod}	N_{cool}	k_s	k_f	C_V^*	*patience*	T_f
$\min u_{20}^{\perp}$	0.03	200	1.5×10^{-5}	$2\,N$	$8\,N$	$30\,N$	2×10^{-9}	10^{-8}	25	10	5×10^{-8}
$\max u_{20}^{\perp}$	0.05	50	2×10^{-5}	$2\,N$	$8\,N$	$30\,N$	5×10^{-9}	10^{-8}	5	10	10^{-7}
$\min u_{00}^{\perp}$	0.02	100	3×10^{-6}	$2\,N$	$3\,N$	$25\,N$	1.6×10^{-9}	1.6×10^{-9}		100	1.5×10^{-8}
$\max u_{00}^{\perp}$	0.05	50	7×10^{-6}	$2\,N$	$8\,N$	$30\,N$	10^{-9}	2×10^{-9}	10	20	3×10^{-8}
$\max u_{00}^{\perp}$ (alt.)	0.03	100	7×10^{-6}	$2\,N$	$3\,N$	$25\,N$	1.4×10^{-9}	2×10^{-9}	2	20	3×10^{-8}
$\Delta\mu_{rel}$	0.03	100	$1.4 \times 1.5^6 \times 10^{-5}$	$2\,N$	$3\,N$	$25\,N$	2×10^{-9}	4×10^{-9}	1	20	$6 \times 1.5^6 \times 10^{-8}$

TABLE I. Parameter settings in our SA algorithm to minimize or maximize $u_{20}^{\perp}$, $u_{00}^{\perp}$, or $\Delta\mu_{rel}$, see the first column. To minimize $u_{00}^{\perp}$, the parameter C_V^* is arbitrary because we set $k_s = k_f$, see Eq. (S2). To maximize $u_{00}^{\perp}$, the parameters in the third row from the bottom are used. Yet, when varying N (see Tab. VII) we use the alternative parameter settings (alt.) of the second row from the bottom.

For elevated numbers of inclusions N, it is necessary to adjust the initial input configuration to fit all inclusions into the sphere when optimizing the magnetostrictive effect. In that case, we use a face-centered cubic (fcc) initial configuration of hexagonal layers oriented perpendicular to the magnetization direction in an ABC stacking configuration. One inclusion is positioned at the center of the elastic sphere. From there, we construct all layers. All nearest-neighbor distances in the fcc configuration approximate the minimal allowed distance. Then, we randomly delete inclusions from the full fcc configuration until the imposed number of inclusions N is reached. For optimizations of the MR effect, we also start with a simple stacking of hexagonal layers of inclusions oriented perpendicular to the magnetization direction. The bottom layer is placed at a distance of $0.061a$ from the lower boundary of the cube and one inclusion is positioned at the center of the layer, with inclusions in different layers placed above each other (denoted by hexagonal in Tab. VIII). Besides, we also utilize fcc arrangements as starting configurations (denoted by fcc in Tab. VIII), similarly to the simple stacking of hexagonal layers, however, with fcc featuring an ABC stacking. (As a remark we add that we never observed a decrease in performance after applying our scheme of optimization when compared to the performance of the initial configuration.)

Furthermore, all optimizations are performed starting from 8 different initializations of the employed random number generator Mersenne Twister [4], leading to comparable results. Deviations in the results are at most of the order of the symbol sizes in the presented figures. Among these, we always selected those configurations of most competitive performance when plotting Figs. 2–7 of the main text.

ν	α
0.5	1
0.3	1.03
0	1.17
-0.5	1.94

TABLE II. Parameter settings in our SA algorithm when minimizing $u_{20}^{\perp}$ for different values of the Poisson ratio ν, leading to the results in Fig. 2D of the main text.

N	α	initialization
1000	1	random
1250	1.25	random
1500	1.5	random
1750	1.5	fcc
2000	1.5	fcc

TABLE III. Parameter settings in our SA algorithm when minimizing $u_{20}^{\perp}$ for different inclusion numbers N, leading to the results in Fig. 2E of the main text.

ν	α	C_V^*
0.5	1	5
0.45	1.01	5
0.4	1.08	5
0.35	1.19	5
0.3	4/3	5
0	2.57	5
-0.5	6.77	2

TABLE IV. Parameter settings in our SA algorithm when maximizing $u_{20}^{\perp}$ for different values of the Poisson ratio ν, leading to the results in Fig. 3D of the main text.

N	α	initialization
1000	1	random
1250	1.25	random
1300	1.3	random
1350	1.35	random
1400	1.4	random
1450	1.45	random
1500	1.5	random
1750	1.5	fcc
2000	1.5	fcc

TABLE V. Parameter settings in our SA algorithm when maximizing $u_{20}^{\perp}$ for different inclusion numbers N, leading to the results in Fig. 3E of the main text.

N	α	initialization
500	0.5	random
650	0.65	random
800	0.8	random
1000	1	random
1250	1.25	random
1500	1.5	random
1750	1.75	fcc
2000	2	fcc
2100	0.995	fcc
2200	0.936	fcc
2300	0.867	fcc
2400	0.788	fcc

TABLE VI. Parameter settings in our SA algorithm when minimizing $u_{00}^{\perp}$ for different inclusion numbers N, leading to the results in Fig. 4C of the main text.

N	α	initialization
1000	1	random
1250	1.25	random
1500	1.5	random
1750	1.75	fcc
2000	2	fcc

TABLE VII. Parameter settings in our SA algorithm when maximizing $u_{00}^{\perp}$ for different inclusion numbers N, leading to the results in Fig. 5C of the main text.

N	α	initialization
150	0.75	random
200	1	random
250	1.25	random
300	1.5	random
350	1.75	random
400	2	hexagonal
450	2.25	hexagonal/fcc (uniaxial elongation, max.)
500	2.5	hexagonal/fcc (uniaxial elongation, max.)

TABLE VIII. Parameter settings in our SA algorithm when maximizing the magnitude of the MR effect $\Delta\mu_{rel}$ for different inclusion numbers N, leading to the results in Figs. 6B,D and Figs. 7B,D of the main text.

II. DETAILS ON THE REGULAR LATTICES TO WHICH WE COMPARE OUR OPTIMIZED STRUCTURES

In the main text, we compared the performance of our optimized configurations to those resulting from more regular arrangements. The latter were constructed artificially, guided by the optimized textures. In the following, we include details on the employed regular structures, first for the studies on magnetostrictive effects, then for the studies on magnetorheological (MR) effects.

A. Regular lattice structures for comparison with optimized magnetostrictive effects

We begin by minimizing $u_{20}^{\perp}$. The performance of our optimized structures in Figs. 2D,E of the main text is compared to results obtained for more regular textures. Specifically, these were regular structures consisting of hexagonally arranged chain-like aggregates. To construct them, we start from layers of hexagonally positioned inclusions. Their normal vectors coincide with the magnetization direction (z-direction). To maximize the magnetic repulsion within each hexagonal layer, we approach the minimal allowed inclusion distance by setting the lattice constant to $0.121R$. The distance between neighboring layers is set to an identical value of $0.121R$. Inclusions within nearest-neighboring layers are located above and below each other, so that they form chains. For simplicity, we anchor the lattices by placing one first inclusion at the center of the sphere and by building up the lattice from there. For a regular arrangement of $N = 1001$ inclusions (to be compared to the optimized structure for $N = 1000$), we confine ourselves to the 11 innermost layers. Moreover, we only consider inclusions that are located within a distance of $\rho = 0.62R$ from that center axis of the sphere oriented parallel to the magnetization direction. The resulting deformations characterized by $u_{20}^{\perp}$ for this configuration and for different values of the Poisson ratio ν are displayed in Fig. 2D of the main text.

To accommodate increasing numbers of inclusions N

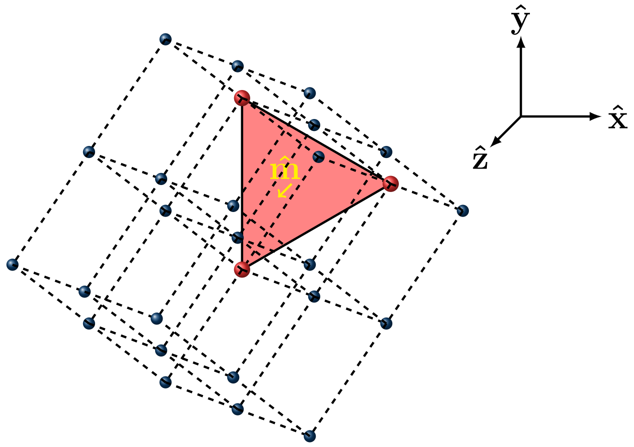

FIG. S1. To best approximate the deformational response of the optimized structure for maximal relative elongation $u_{20}^{\perp}$, we identified a simple cubic lattice. Here, we illustrate its orientation. The normal vector of the plane indicated in red coincides with the magnetization direction $\hat{\mathbf{m}}$ (by construction).

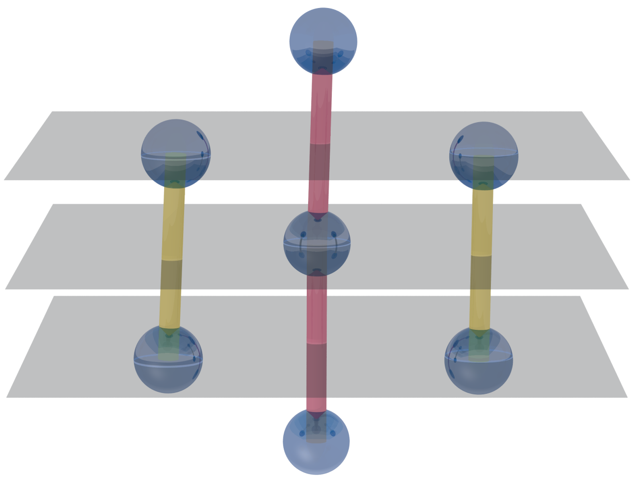

FIG. S2. Configurations that maximize the decrease in volume, minimizing $u_{00}^{\perp}$, see Fig. 4A of the main text, contain chain-like elements along the magnetization direction. Here, we illustrate the typical nearest-neighbor structure of these chains, where inclusions in neighboring chains are vertically shifted relative to each other by about one half (vertical) nearest-neighbor distance.

we increase ρ. Moreover, we slightly shift the regular arrangements of inclusions homogeneously along the magnetization direction by a distance Δz. We observe that the optimized structures do not feature a layer exactly in the middle of the sphere. Indeed, through numerical trials with different values of Δz the performance was increased further. Appropriate numbers n_+ and n_- of layers above and below the center of the sphere, respectively, are determined by numerical trials as well to further optimize the deformational response. For $N = 1001$, 1308, 1482, 1771, 1988, we use values of $\rho/R = 0.62$, 0.65, 0.73, 0.78, 0.84, $\Delta z/R = 0$, 0.043, 0.019, 0.005, 0.019, $n_+ = 5$, 6, 6, 7, 7, and $n_- = 5$, 6, 6, 6, 7, respectively. Corresponding results for $u_{20}^{\perp}$ are shown in Figs. 2D,E of the main text.

Next, when maximizing $u_{20}^{\perp}$ (see Fig. 3 of the main text), we identified a simple cubic (sc) lattice to best approximate the optimized structures. As in the previous case, a lattice constant of $0.121R$ is employed. The orientation of this regular structure provides additional degrees of freedom to improve its performance. We start from an sc lattice for which the lattice vectors coincide with the coordinate axes of our Cartesian coordinate system, the z-direction again indicating the magnetization direction. Then, a global rotation is applied to this lattice. We found a rotation by a rotation matrix

$$\underline{\mathbf{R}} = \begin{pmatrix} \sqrt{\frac{2}{3}} & -\sqrt{\frac{1}{6}} & -\sqrt{\frac{1}{6}} \\ 0 & \sqrt{\frac{1}{2}} & -\sqrt{\frac{1}{2}} \\ \sqrt{\frac{1}{3}} & \sqrt{\frac{1}{3}} & \sqrt{\frac{1}{3}} \end{pmatrix} \quad \text{(S3)}$$

to approximately lead to best performance. It sets a rotation by a rotation angle of about 56.6° around the axis $\big((\sqrt{3}-1)(\sqrt{2}-1)+1,\ -1,\ \sqrt{2}-1\big)$. The resulting orientation is illustrated in Fig. S1.

We again place the first inclusion at the center of the sphere. Furthermore, to set the number of inclusions, we only include lattice positions within a distance r from the center. We use $r/R = 0.747$, 0.81, 0.82, 0.83, 0.85, 0.86, 0.88, 0.9, 0.94 to obtain $N = 1021$, 1237, 1309, 1357, 1419, 1503, 1575, 1743, 1935, respectively. The resulting performance for $u_{20}^{\perp}$ is depicted in Figs. 3D,E of the main text. In the latter figure, we also display results for a face-centered cubic (fcc) configuration of $r/R = 0.84$. There, the edges of the cubic unit cells are oriented along the axes of our Cartesian coordinate system, leading to $N = 1985$. In that case, we likewise use a nearest-neighbor distance of $0.121R$.

Third, we optimize $u_{00}^{\perp}$, starting with its minimization, see Fig. 4C of the main text. In that case, we use regular chain-like structures with a vertical shift of neighboring chains by half the inter-inclusion distance along each chain, as was similarly observed for the optimized structures, see Fig. S2. The lateral arrangement of these chains in the optimized structure is mostly irregular. For our regular structures, we arrange the chains in a square lattice. The corresponding lattice constant is set to approximately $0.108R$. We construct the configuration starting from a chain that runs through the center of the sphere. The center is located symmetrically between the two innermost inclusions on that chain. As in the previous case, we use a radial cut-off distance r from the center of the sphere to adjust the number of inclusions N. We set $r/R = 0.55$, 0.6, 0.65, 0.7, 0.75, 0.8, 0.84, 0.878, 0.89, 0.907, 0.92, 0.932 to obtain $N = 494$, 654, 798, 1024, 1240, 1502, 1742, 2006, 2094, 2206, 2296,

2392, respectively.

Next, we compare the results for textures of maximized $u_{00}^{\perp}$, see Fig. 5C of the main text, to the performance of regular configurations of inclusions. To this end, we arrange the inclusions in 11 layers of orientation normal to the magnetization direction. Within each layer, the inclusions are hexagonally organized, with a distance between nearest neighbors of $0.121R$. The textures are constructed starting from one inclusion at the center of the sphere. In this case, however, the inter-layer spacing l is always chosen larger than $0.121R$. Thus, nearest neighbors are found within the layers, not along the magnetization direction. By adjusting l and only keeping inclusions that are located within our constraints inside the sphere, we change the number of inclusions N. Setting $l/R = 0.27$, 0.22, 0.18, 0.155, 0.126 leads to $N = 1021$, 1239, 1517, 1745, 1973, respectively.

B. Regular lattice structures for comparison with optimized magnetorheological effects

Similarly, we construct by hand regular lattice structures, the performance of which we compare to our optimized ones that maximize the MR effect as described in the main text for an elastic cubical system. In general, the optimized structures guide us to the particular parameters used for our constructed regular lattice structures. In this way, the latter are adapted to the former.

For the positive MR effect, maximizing $\Delta\mu_{rel}$ for uniaxial elongation, see Figs. 6A,B of the main text, we utilize an fcc arrangement (ABC stacking of hexagonal layers) with a lattice constant of $0.121a$, where a is the side length of the elastic cube. The bottommost hexagonal layer when viewed along the magnetization direction is always inserted at $0.1a$ from the bottom surface of the cube. The lattice is anchored by placing an inclusion at the center of the cube. A cut-off value ρ for the lateral distance of an inclusion from the center axis of the cube oriented parallel to the magnetization direction is set to tune the number of inclusions. Here, the value of ρ is chosen per layer, adapted from the optimized structures, generally decreasing the numbers of inclusions in the topmost and bottommost layers when compared to the other layers. For example, for $N = 201$ inclusions (for comparison to the optimized structures of $N = 200$), we use $\rho = 0.2a$ for the topmost and bottommost layers and $\rho = 0.35a$ for another 6 layers in between, resulting in a total of 8 layers. We also generate fcc configurations of 255, 300, 354, 399, 449, 497 inclusions, here always using 9 layers of varying ρ. The layer distance is chosen such that an fcc lattice of lattice constant $0.121a$ results.

When instead maximizing the positive MR effect for simple shear deformations, see Figs. 6C,D of the main text, textures consisting of hexagonally structured layers with their normals oriented along the direction of the shear displacements are introduced. The same lattice constant as above is used. This time, for each layer an inclusion is placed on that center axis of the cube that is oriented along the direction of shear displacements. The number of layers and locations of layers are adapted from the optimized configurations. We alternately select opposite sides from one layer to the next with respect to the central shear plane. Approximately, our procedure consists of cutting inclusions from these sides, starting from maximal distance from the central shear plane. We observe such asymmetric features in the optimized structures, see Fig. 6D of the main text. This procedure leads to $N = 187$, 291, 347, 402, 448, 499 inclusions organized in 4, 5, 6, 6, 7, 8 layers, respectively. In the last two cases, we use an alternating AB stacking of the layers to fit all inclusions into the cube.

Turning to the less frequently considered negative MR effect, we first consider the case of uniaxial elongation of the cube in the main text, see Figs. 7A,B. Neighboring chains forming when optimizing the structures are vertically shifted with respect to each other, similarly to what is displayed in Fig. S2. We generally use a square lattice arrangement of these chains for simplicity, as its performance shows good agreement in the resulting negative MR effect. For comparison, for $N = 180$ we also arrange 24 chains on a circle of radius $0.42a$, showing a magnitude of the negative MR effect in line with the trend of corresponding square lattice arrangements of the chains. We shift the inclusions in neighboring chains vertically by half of the nearest-neighbor distance in a single chain ($0.121a$). Their lateral distance is chosen such that neighboring inclusions from different chains have the same total distance from each other of $0.121a$. Therefore, each inner inclusion on a chain has two nearest neighbors on the same chain and two more per neighboring chain, for a total of up to 10 nearest neighbors. To tune the inclusion numbers of the square lattice arrangements of the chains, we selectively delete chains, leading to arrangements of $N = 150$, 203, 248, 301, 346, 398, 450, 503 with 20, 27, 33, 40, 46, 53, 60, 67 chains, respectively. The chains for deletion are selected by comparison to the optimized structures, which are displayed as the insets in Fig. 7B of the main text, approximately such that the square lattice of chains has a gap in the middle. Results for these square lattice arrangements as well as the one circular arrangement of the chains mentioned above for $N = 180$ are presented in Fig. 7B of the main text (chain-like config.). The data point for $N = 180$ of circular arrangement of the chains is highlighted by the black circular mark.

Lastly, we turn to the negative MR effect under simple shear deformations, see Figs. 7C,D of the main text. The resulting optimized structures in that case are similar to the ones for the positive MR effect under simple shear. However, the layers here are oriented parallel to the plane of simple shear. Furthermore, as can be inferred from Fig. 7C of the main text, the layers do not consist of regular hexagons anymore. Instead, they resemble a centered rectangular lattice, with the longer edge along the magnetization direction. We choose $0.146a$ for the edge length along the direction of shear displacements and

0.194a for the edge length along the magnetization direction. Again, we set the numbers and locations of the layers as inferred from the optimized structures. To restrict the number of inclusions in each layer, we use a cut-off value ρ for the lateral distances of the inclusions from that center axis that is oriented perpendicular to the shear plane, similar to the maximization of $\Delta\mu_{rel}$ for uniaxial elongation (in contrast to the rather diagonal reduction in Fig. 7C of the main text). Here, ρ is chosen for each layer again by comparison to the optimized structures. For example, we observe that the optimized structures for $N = 200$ inclusions feature a center layer (see the leftmost inset of Fig. 7D of the main text) with fewer inclusions compared to the other layers. Therefore, we choose a smaller value for ρ for that layer. In this way, we obtain $N = 203$, 248, 297, 347, 399, 450, 497 with 5, 6, 7, 7, 7, 8, 9 layers, respectively. For $N = 497$, we use an alternating AB stacking of the layers.

[1] M. Karabin and S. J. Stuart, Simulated annealing with adaptive cooling rates, J. Chem. Phys. **153**, 114103 (2020).

[2] N. Metropolis, A. W. Rosenbluth, M. N. Rosenbluth, A. H. Teller, and E. Teller, Equation of state calculations by fast computing machines, J. Chem. Phys. **21**, 1087 (1953).

[3] S. Kirkpatrick, C. D. Gelatt, and M. P. Vecchi, Optimization by simulated annealing, Science **220**, 671 (1983).

[4] M. Matsumoto and T. Nishimura, Mersenne Twister: a 623-dimensionally equidistributed uniform pseudo-random number generator, ACM T. Model. Comput. S. **8**, 3 (1998).
